# Free and open-source software for object detection, size, and colour determination for use in plant phenotyping

**DOI:** 10.1186/s13007-023-01103-0

**Published:** 2023-11-15

**Authors:** Harry Charles Wright, Frederick Antonio Lawrence, Anthony John Ryan, Duncan Drummond Cameron

**Affiliations:** 1 Department of Chemistry, The University of Sheffield, Sheffield, S3 7HF UK; 2 Department of Chemistry, Imperial College London, London, SW7 2AZ UK; 3 Department of Earth and Environmental Sciences and Manchester Institute of Biotechnology, The University of Manchester, John Garside Building, Manchester, M1 7DN UK

**Keywords:** Colour, Chlorophyll, Lycopene, FOSS, Object detection, Open-source

## Abstract

**Background:**

Object detection, size determination, and colour detection of images are tools commonly used in plant science. Key examples of this include identification of ripening stages of fruit such as tomatoes and the determination of chlorophyll content as an indicator of plant health. While methods exist for determining these important phenotypes, they often require proprietary software or require coding knowledge to adapt existing code.

**Results:**

We provide a set of free and open-source Python scripts that, without any adaptation, are able to perform background correction and colour correction on images using a ColourChecker chart. Further scripts identify objects, use an object of known size to calibrate for size, and extract the average colour of objects in RGB, Lab, and YUV colour spaces. We use two examples to demonstrate the use of these scripts. We show the consistency of these scripts by imaging in four different lighting conditions, and then we use two examples to show how the scripts can be used. In the first example, we estimate the lycopene content in tomatoes (*Solanum lycopersicum*) var. Tiny Tim using fruit images and an exponential model to predict lycopene content. We demonstrate that three different cameras (a DSLR camera and two separate mobile phones) are all able to model lycopene content. The models that predict lycopene or chlorophyll need to be adjusted depending on the camera used. In the second example, we estimate the chlorophyll content of basil (*Ocimum basilicum*) using leaf images and an exponential model to predict chlorophyll content.

**Conclusion:**

A fast, cheap, non-destructive, and inexpensive method is provided for the determination of the size and colour of plant materials using a rig consisting of a lightbox, camera, and colour checker card and using free and open-source scripts that run in Python 3.8. This method accurately predicted the lycopene content in tomato fruit and the chlorophyll content in basil leaves.

**Supplementary Information:**

The online version contains supplementary material available at 10.1186/s13007-023-01103-0.

## Background

Quantitative colour analysis is an important aspect of plant material phenotyping and adds valuable information in a variety of common applications. Colour has been shown to be an accurate predictor of ripeness in a variety of fruits, including bananas [1], tomatoes [2], and avocados [3]. As fruit ripens, the ratios of coloured compounds such as carotenoids, anthocyanins, and chlorophyll change and colourimetric methods are routinely used to determine the concentration of these compounds, which can provide important information about the quality and nutrition content of the plant/fruit [4]. Colour, in addition to fruit ripeness, is a good predictor of chlorophyll concentration in plant leaves, and models for several plant varieties, including rice [5], quinoa and amaranth [6], and *Arabidopsis* [7], have been developed. Furthermore, colour can be used to detect plant disease, which can save considerable time in plant pathological analysis [8].

When non-destructive colorimetric methods are required, visible imaging can be used to measure plant phenotypes such as leaf area, colour, yield, disease severity, fruit number, etc., and digital photography is the easiest and cheapest of these methods [9]. Many pipelines and tools have been developed for determining the size or colour of plant tissues; however, they often require the use of closed-source software/tools or extensive manual image correction [6, 10]. Eliminating proprietary software and developing free and open source software (FOSS) has many benefits, including reducing research costs and generating software that is highly suitable for the required scientific task [11, 12] and makes these techniques available to researchers with limited resources.

An improvement to FOSS that increases transparency, reproducibility, and reusability of software is to follow the “FAIR” principles by producing software that is Findable, Accessible, Interoperable and Reusable, which, when used specifically for research software, is known as FAIR4RS [13]. Some recommendations have been made for FAIR4RS, including ensuring users know how to retrieve and cite software for findability, ensuring software is preserved via “snapshots” using online repositories with concurrent version control to tag new releases to improve accessibility; adhering to software standards for interoperability; and including adequate documentation in conjunction with test data for improving reproducibility [14]. Following FOSS and FAIR principles allows for the creation of plant colour software that is transparent, robust, and repeatable. This kind of colour detection system allows for generalisation of findings between systems and can help improve models used for colour detection and characterisation [15].

In this manuscript, we present a pipeline and set of FOSS scripts that follow the FAIR4RS principles for colour correcting digital photo images, separating objects, and determining the objects' size and colour coordinates in various colour spaces. The pipeline is tested in four different lighting conditions to show the consistency of the colour correction pipeline. Two examples are then used to demonstrate possible use cases for these scripts: first, determining the lycopene content of tomatoes, which is done using three different cameras; and second, determining the chlorophyll content of basil leaves.

## Materials and methods

### Materials

1. Any digital camera (whilst a DSLR camera that shoots in RAW is preferable, mobile phone cameras can also be used).
2. A 24 swatch colour checker (code provided and colour file are for SpyderCheckr24, https://spyderx.datacolor.com/shop-products/)
3. A lightbox with supplementary lighting (this is optional however it does improve the quality of the results, see for example https://tinyurl.com/s8x4v2jd). The example lightbox contains LED lighting; this could be further improved by using bulbs that are closer to standard illuminants (D65 for sRGB).
4. An object of known size (coins work well).
5. Software: Python 3.8.
6. Python packages: List of packages and their versions used available in Additional file 1: S0.
7. Custom Python Scripts: https://github.com/HarryCWright/PlantSizeClr
8. Snapshot of all scripts and data is available on Open Science Framework: www.doi.org/10.17605/OSF.IO/QAYMU
9. Optional for extraction of lycopene: acetone, high purity ethanol, hexane deionised water and a UV/vis spectrophotometer
10. Optional for extraction of chlorophyll: 80% acetone in deionised water and a UV/vis spectrophotometer

### Protocol

**Fig. 1:**
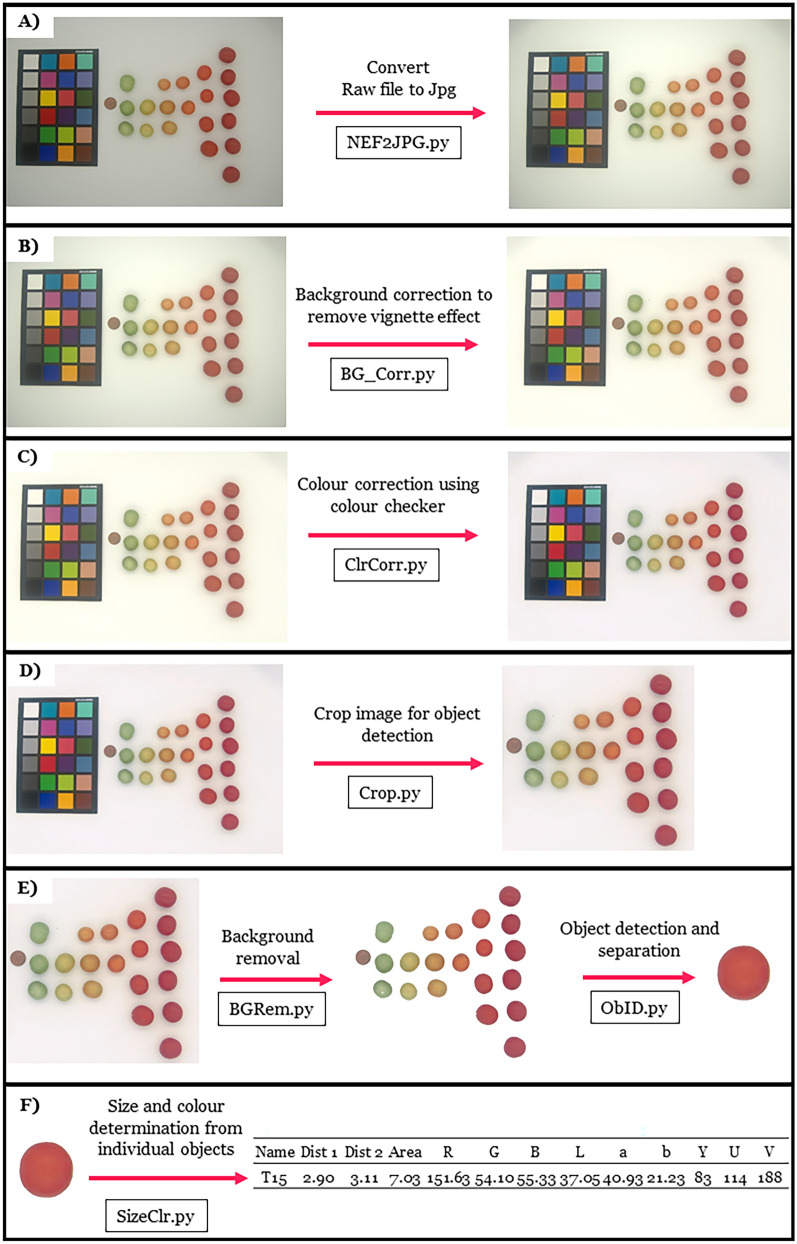
The framework for determining the size and colour of objects for plant phenotyping. The steps consist of (**A**) converting the raw file into a jpg file (optional) using the script NEF2JPG.py, (**B**) doing a background correction to account for the vignette effect by the camera lens using the BG_Corr.py file, (**C**) colour correction using a colour checker card using ClrCorr.py, (**D**) cropping of image for object identification using Crop.py, (**E**) removal of the white background using BGRem.py (**F**) separation of objects using ObID.py and (**G**) determination of the size and colour of objects using SizeClr.py

Figure 1A–F shows the general protocol and pipeline using the presented scripts for image correction, object detection and separation and colour and size determination and in depth details are provided.

1. A background image should be taken with the digital camera to perform a background correction that corrects for any lightness uniformity due to the camera sensor or inhomogeneous lighting conditions. An example of a background image is provided in Additional file 2: S1.
2. Photograph plant tissue using a digital camera, ensuring that plant tissue, colour checker board as well as an object of known size are all visible in the photo. Background removal and object separation relies on using a white background so users should capture images with a white background. The colour checker should be in a portrait orientation and the object for calibrating size should be the left most object (other than the colour checker board) an example of a good image is given in Fig. 1 A and in more detail in Additional file 3: S2. The scripts may not identify the colour checker if the colour checker is not square in the image, it is important to ensure it is when capturing the images, otherwise the image may need to be cropped and rotated such that the colour corrector is square.

Note 1: Software scripts function for photos taken in both landscape and portrait, if using the SpyderChecker24 colour checker, the light blue swab should be in the top right and portrait irrespective of the image orientation.

Note 2: Photographs can be taken in varying lighting conditions; however, the use of lighting conditions that best imitate standard CIE illuminants (D55 or D65) will result in images with the lowest colour error. It is important to ensure that there are no shadows cast onto the image or projected from the plant tissues, as this affects colour correction and object separation.

Note 3: All objects of interest (colour checker board, object of known size, plant tissue) should be placed as near to the centre of the photo as possible to minimise lens distortion effects.

3. If images are captured in RAW format, they need to be converted to *.jpg format, if a Nikon camera is used that saves RAW files as *.NEF file, then the included script NEF2JPG.py can be used to convert the file to jpg. This script can batch process all the files in a folder. If RAW images from other devices are used they will need to be converted to jpg outside of this pipeline before continuing with step 4.
4. Background correction is done on the *.jpg files. The script BG_Corr.py is able to batch process all files in a particular folder. The script will prompt you to select the background image file captured for background correction. It will then ask you for the folder with all the images that required background correction before corrected the files. Files will be saved using their initial filename plus”_BGcorrected.jpg”.
5. Colour correction is done on background corrected jpg files. The script will prompt you for the *.csv file that contains the RGB data of your colour correction swatches (if using SpyderCheckr24 this is supplied in Additional file 4: S3). The order of the colour swatches entered into the.csv file is important and Additional file 5: S4 demonstrates the order the values need to be entered into the file. It will then ask you for the folder that contains the images that you wish to correct and proceed to colour correct the batch of images. It will print out the r [2] values of the colour correction matrix and these should all be above 0.95 if images have been successfully corrected. Corrected images will be saved using their initial filename plus the suffix “_fin.jpg”. Furthermore a.csv file will be generated that saves the average swatch error for each file before and after colour correction. This function also prints and saves the RGB values of the white swatch, to ensure that colour correction is not resulting in saturated RGB values. A note is printed in the command line stating whether there may be saturation problems that need further investigation.
6. For object identification and separation it is necessary to crop the colour corrector out of the colour corrected images as shown in Fig. 1D. The provided script Crop.py is able to do this cropping. It will prompt you to select the folder with the corrected images and then display the images one at a time. The user must click and drag a rectangle on the image with their mouse to select the region of interest (ROI). The region of interest should include all objects of interest as well as the object of known size. The object of known size should now be the left most object in the image. Once the user is happy with the ROI they press enter and this will crop and save the image and open the following image. Images will be saved as their initial filename plus the suffix “_crop.jpg”.
7. Background removal is done using the script BGRem.py. The user will be prompted to select the folder that has the images which require background removal. This script also has a threshold value that can be changed if the background removal is either cropping the objects of interest by being too aggressive in thresholding or not removing the background entirely. This is changed on line 16 of the script BGRem.py (a good starting value is 150). Images with their background removed are saved as their initial name plus the suffix “_BGRem.jpg”.
8. Object separation is done using the script ObID.py which will prompt the user to select the folder which has the image files for object separation. Once the script is run, an image will be displayed with an identified object highlighted and the user will be asked to give this object a name in the IDE software used to run the script. The object will then be saved as a jpg file with this name. This will then repeat for the next object in the image and once all objects have been named the script will move to the next image in the folder.
9. Finally colour and size information can be extracted from the individual saved objects. This is done using the script SizeClr.py. This will prompt the user for the folder with the separated object images and for the file that contains the object of known size. The user will be prompted for the height and width of the object in the unit of interest and should enter this in the IDE. The script will then work through each of the objects and extract their size data (width, height and area) as well as the colour in three colour spaces; RGB, YUV and CIELAB. For CIELAB it is assumed that the observer is 2° and the illuminant is a CIE standard illuminant D65. If this is not the case the user should change these values on line 583–585 of script PlantSzClr.py. Colour and size information is saved in a new.csv file. The user will be prompted to give the name of this csv file and information is saved in columns as shown in Fig. 1F.

### Image processing

Although detailed understanding of the backend processing is not required to use the pipeline, some understanding can benefit the user, particularly for background correction, colour correction, object separation, and colour determination.

Background correction: This function corrects an experimental image using a background image captured under the same conditions and camera settings. First, the background image is blurred (5 × 5 pixels) and normalised to values between 0 and 1. Then, the experimental image is divided by the normalised background image to correct for any differences in lighting and exposure. The resulting corrected image is clipped to the valid range (0,255) and saved.

**Fig. 2:**
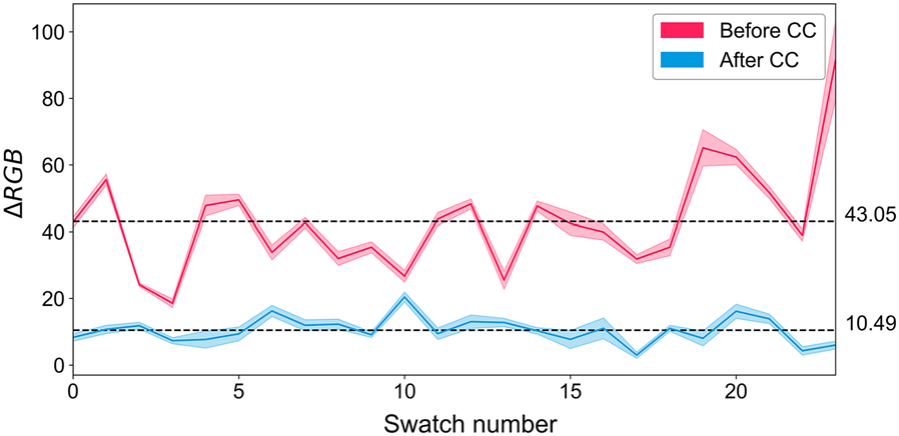
The error between captured RGB values and the sRGB (ΔRGB) values for the 24 swatches on the SpyderCheckr24 colour checker before and after colour correction. The filled area indicates the standard deviation (n = 50) for different images of lettuces captured in a light box. The mean error before and after colour correction is shown on the right hand side

Colour correction: A colour correction process was performed using the colour_checker_detection python package with slight modifications to ensure accurate identification of all 24 swatches. The function implements polynomial regression to adjust for any discrepancies between the observed and reference RGB values of the swatches. The process takes in two parameters: an array of the reference sRGB values for the 24 swatches and the observed mean RGB colours of the same swatches. The function creates a design matrix with the observed RGB values and their powers up to the third order and fits a linear regression model to each of the R, G, and B channels using this matrix and the swatch sRGB values. The optimal correction coefficients were determined through ordinary least squares regression. The RGB channels of the experimental image were corrected by applying the correction transformation (Eq. 1).

1 $${\text{CC}}_{{{\text{Corr}}}} = \upbeta_{{1,{\text{CC}}}} {\text{R}}_{{\text{i}}} + \upbeta_{{2,{\text{CC}}}} {\text{G}}_{{\text{i}}} + \upbeta_{{3,{\text{CC}}}} B_{{\text{i}}} + \upbeta_{{4,{\text{CC}}}} {\text{R}}_{{\text{i}}}^{2} + \upbeta_{{5,{\text{CC}}}} {\text{G}}_{{\text{i}}}^{2} + \upbeta_{{6,{\text{CC}}}} {\text{B}}_{{\text{i}}}^{2} + \upbeta_{{7,{\text{CC}}}} {\text{R}}_{{\text{i}}}^{3} + \upbeta_{{8,{\text{CC}}}} {\text{G}}_{{\text{i}}}^{3} + \upbeta_{{9,{\text{CC}}}} {\text{B}}_{{\text{i}}}^{{3}}$$

where CC_corr_ is the corrected colour channel (R, G or B) value, β is the correction coefficient for each channel, and R_i_, G_i_ and B_i_ are the observed red, blue and green values within the image. This type of polynomial regression has been successfully used to minimise error when transforming from RGB to device-independent sRGB that minimises the error due to non-ideal illuminants. Polynomial modelling has also been used to convert directly to XYZ tristimulus values [16]. The sRGB colour space also has a relationship to the CIE colourimetric colour space and allows for conversion between these colour spaces [17]. To demonstrate the error reduction 50 images of lettuce were captured in a lightbox and the average error between the reference swatch sRGB value and captured value was calculated according to Eq. (2).

2 $$\Delta {\text{RGB}}_{{\text{i}}} = \sqrt {({\text{R}}_{{{\text{ref}},{\text{i}}}} - {\text{R}}_{{\text{i}}} )^{2} + ({\text{G}}_{{{\text{ref}},{\text{i}}}} - {\text{G}}_{{\text{i}}} )^{2} + ({\text{B}}_{{{\text{ref}},{\text{i}}}} - {\text{B}}_{{\text{i}}} )^{2} }$$

where R_ref,i_, G_ref,i_ and B_ref,i_ are the reference sRGB values for the three colour bands for each of the swatches, R_i,_, G_i,_ and B_i_ are the observed colour band values in the image and ΔRGB_i_ is the error for each of the 24 swatches. The mean error of the 24 swatches over the fifty images before colour correction was 43.05 and this was reduced to 10.49 once colour correction had taken place (Fig. 2).

Background removal: This function removes the background of an image using colour thresholding and morphological transformations (cv2.getStructuringElement) and cv2.morphologyEx) which exclude small pixels and remove all except large objects.

Object separation: This function is designed to perform object detection and segmentation on digital images. It employs Canny edge detection to extract edges from the input image and applies contour detection to identify and sort the contours in the resulting edge map. The function then computes the mean RGB color of each object and transforms these values into YUV and CIE L*a*b* color bands. To determine the object’s maximum width and height, as well as its area, the function counts the number of non-white pixels***.***

### Note on ideal plant tissue

Although there are many applications for this type of colorimetric determination in plant phenotyping, it must be noted that for the two case studies provided the plant tissues are ideal for this type of phenotyping (basil leaves are smooth, without wax or hairs, green and unvariegated and tomatoes follow a green/yellow/orange/red ripening process and are undamaged and free from dirt). For non-ideal leaves there can be a significant change in their reflectance patterns and intensity [18] which would likely need a more complex pipeline for understanding colour than the pipeline provided here as would damage or dirty tomato fruit.

### Lycopene extraction

Lycopene extraction was done as a case study using the pipeline. The lycopene was solvent extracted and its concentration measured by UV/vis spectrophotometry. Lycopene extraction was completed according to the method described by Fish et al*.* [19] with a slight modification. Fresh tomato tissue was finely ground in a mortar and pestle with an equal weight of deionised water to give a paste. 0.4–0.6 g of this paste was added to a vial and kept on ice in the dark until processing. To each vial, 5 mL of acetone, 5 mL of 95% ethanol, and 10.0 mL of hexane were added. Vials were sealed and placed on their sides in a container that contained ice, and they were mixed for 15 min at 180 rpm on an orbital shaker. After shaking, 3 ml of deionised water was added to the vials, and they were shaken for another 5 min. Samples were then left to phase separate at room temperature for 5 min before absorbance was measured. The UV/vis absorbance of the upper (hexane) fraction was measured between 400 and 700 nm, and the intensity at 503 nm was used for lycopene content determination, measured against a hexane blank. The lycopene concentration was calculated according to Eq. (3).

3 $${\text{L = }}\frac{{{\text{A}}_{{{503}}} }}{{{17}{\text{.2 }} \times {10}^{{4}} {\text{M}}^{{ - 1}} {\text{cm}}^{{ - 1}} { }}} \times \frac{{{536}{\text{.9 g}}}}{{{\text{mole}}}} \times \frac{{\text{1 l}}}{{{10}^{{3}} {\text{ml}}}} \times \frac{{{10}^{{3}} {\text{ mg}}}}{{\text{g}}} \times \frac{{\text{10 ml}}}{{\text{kg tissue}}}{ = }\frac{{{\text{A}}_{{{503}}} \times \;31.2}}{{\text{g tissue}}}$$

where L is the lycopene concentration in mg kg^−1^, A_503_ is the absorbance at 503 nm, 17.2 × 10^4^ M^−1^ cm^−1^ is the molar extinction coefficient for lycopene in hexane [20].

### Lycopene model training and validation

A large range of models have been suggested for fitting colour data from digital images to lycopene concentration, including linear and exponential models. Liñero et al. used a quadratic equation using the CIELAB colour space, which explained 95 percent of the variance [21], whereas Arias et al. used linear and exponential fits using parameters from the CIELAB colour space to classify tomatoes into maturity groups and to predict lycopene content. An exponential model in the form shown in Eq. (4) was found to be the best predictor of lycopene concentration, with an r^2^ of 0.96. [22].

4 $${\text{L}}_{{{\text{pred}}}} { = }\upbeta_{{1}} {\text{e}}^{{\upbeta_{{2}} \frac{{{\text{a}}^{*} }}{{{\text{b}}^{*} }}}} { + }\upbeta_{{3}}$$

where L_pred_ is the predicted lycopene content, β_1_, β_2_ and β_3_ are fitting parameters and a^*^ and b^*^ are colour channels in the CIELAB colour space. For this case study Eq. (4) is used to fit lycopene data.

### Chlorophyll extraction

Chlorophyll extraction was used to provide a second example of the pipeline. Chlorophyll was extracted from basil leaves, and the chlorophyll content was determined using UV/vis spectrophotometry. 70−100 mg of fresh leaf matter was ground to a paste with a pestle and mortar in 3 ml of an 80% acetone solution. This turbid paste was transferred to a 15 ml centrifuge tube. A further 1.5 ml of 80% acetone was used to rinse the mortar and pestle, and the final solution was brought up to 5 ml with 80% acetone [23]. Chlorophyll extraction was allowed to take place in the dark overnight. Vials were then centrifuged at 4000 RPM for 10 min, and the supernatant was collected for spectrophotometry. The UV/vis absorption at 646 nm and 663 nm was used to estimate the chlorophyll content according to Eqs. (5, 6 and 7) [24].

5 $${\text{C}}_{\text{a}}\text{ = }{\text{12.21A}}_{663}-{\text{2.81A}}_{646}$$

6 $${\text{C}}_{\text{b}}\text{ = }{\text{20.13A}}_{646}-{\text{5.03A}}_{663}$$

7 $${\text{C}}_{\mathrm{t}}\text{ = }{\text{C}}_{\text{a}}+{\text{C}}_{\text{b}}$$

where C_a_ is the amount of chlorophyll a, C_b_ is the amount of chlorophyll b and C_t_ is the amount of total chlorophyll (all in µg ml^−1^). A_663_ and A_646_ is the absorption at specific wavelengths.

### Chlorophyll model training and validation

For model fitting of chlorophyll content an exponential model using the RGB colour system has been shown to accurately predict chlorophyll content in several crops including quinoa and amaranth [6], and *Arabidopsis* [7] and is used in case study example. Chlorophyll content was determined using the model shown in Eq. (8).

8 $${\text{Ct}}_{{{\text{pred}}}} {\text{ = e}}^{{\left( {\upbeta_{{1}} {\text{R + }}\upbeta_{{2}} {\text{G + }}\upbeta_{{3}} {\text{B + }}\upbeta_{{4}} } \right)}}$$

where Ct_pred_ is the predicted total chlorophyll content, β_1_, β_2,_ β_3_ and β_4_ are fitting parameters and R, G and B are the colour bands in the RGB colour space.

## Results and discussion

### Lighting test

To determine the flexibility and robustness of the pipeline, and in particular the background and colour correction scripts, images containing just the colour corrector were captured in four different locations with varying lighting conditions. Location one was the most controlled in a LED-lit lightbox, location two was done in natural lighting conditions; and locations three and four were done in two separate laboratories with fluorescent lighting (Light intensity and spectrums given in Additional file 6: S5 measured with a LI-COR LI-180). Images were captured with a Nikon D60 with the following settings: aperture = f/4.2, shutter speed = 1/200 s, ISO = 100, file storage = RAW. The average error of the swatches of the colour corrector, as defined in Eq. (2), was used to determine the deviation of the image from the reference sRGB colour space, and these values were compared for the four locations before and after background and colour correction.

**Fig. 3:**
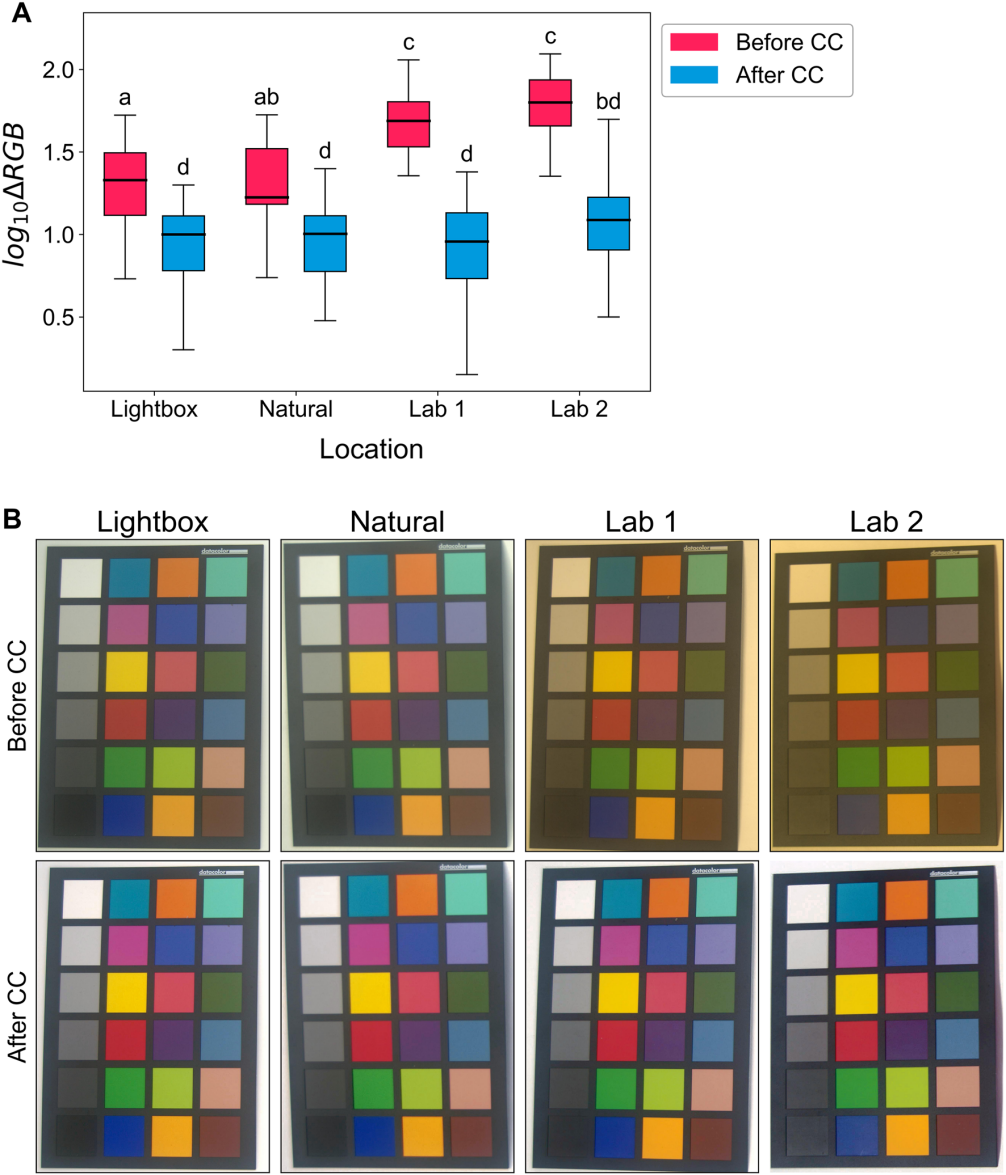
**A** Log_10_ transformations of the error of the 24 colour corrector swatches before colour correction (pink) and after colour correction (blue) for images captured on a Nikon D60 at four different locations with different lighting conditions. The box is created from the first to the third quartile and the horizontal line through the box indicates the median. Thin whiskers show 1.5 × IQR from the edges of the box. Different letters indicate that means are significantly different (Tukey HSD, *p* < .05). **B** Shows the photos of the colour corrector board in the four locations, before and after colour correction

A two-way ANOVA was performed to analyse the effect of location and colour correction on the ΔRGB error. Main effects analysis showed that both location (F(3, 184) = 15.7, *p* < 0.001) and colour correction (F(1, 184) = 221, *p* < 0.001) had significant effect on the ΔRGB error. A statistically significant interaction between the effects was also revealed by the two-way ANOVA (F (3, 184) = 9.57, *p* < 0.001) (Table 1). The light box and naturally lit images had statistically significantly lower error than the two laboratories with fluorescent lighting, which had higher error before colour correction (Fig. 3A). For all locations, the ΔRGB error reduced after colour correction, and, after colour correction the error did not differ between the four locations (Fig. 3A). The log10 transformed data is presented in Fig. 3 A as tests for normality and equal variance were not met with untransformed data. Additional file 7: S6 displays the untransformed error data. The images on the colour correction card make visualising these errors before and after colour correction easier, and these images are shown in Fig. 3B. The mean untransformed mean ΔRGB error for the four locations (lightbox, natural, lab 1 and lab2), after colour correction were: 10.6, 10.4, 10.0 and 14.3 (Additional file 7: S6) with the first three having similar error to that shown in Fig. 2.

**Table 1:** Two-way ANOVA table for the error of swatches on colour corrector card

Factor	SS	df	F	PR(> F)
Location	2.98	3	15.7	< 0.001
Corrected	14.0	1	221	< 0.001
Location x corrected	1.82	3	9.57	< 0.001
Residual	11.6	184	–	–

This analysis reveals that although the lighting conditions have a large influence on the ΔRGB error, once colour correction is done, the error is comparable between locations. The pipeline can be used in varying lighting conditions; however, it should be noted that locations with more consistent controllable light (natural and lightbox) had the lowest errors before colour correction and produced more consistent images. It is suggested that a lightbox be used in conjunction with this pipeline; however, in situations where this is not possible, using supplementary daylight LEDs (5000–6500 K) can increase consistency and decrease ΔRGB error.

### Lycopene extraction

**Fig. 4:**
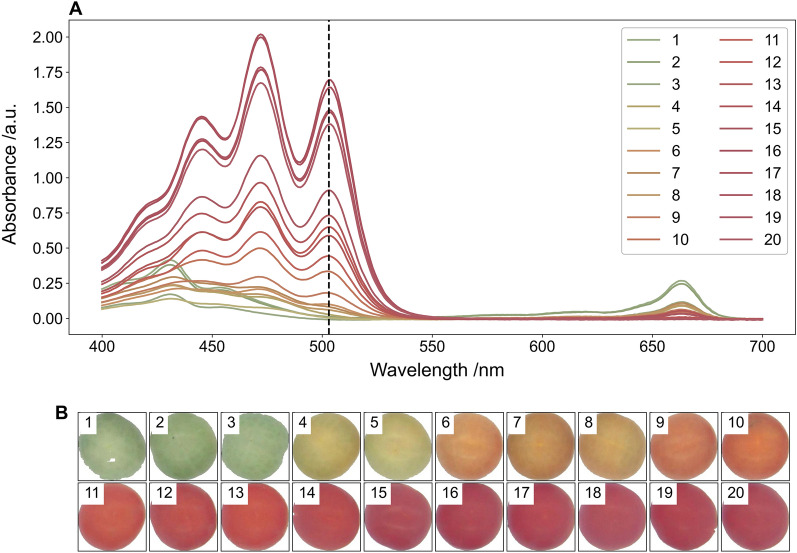
**A** UV spectrophotometry spectrum for tomato fruit with the vertical dashed line indicating the wavelengths used to determine lycopene content (503 nm) and **B** background and colour corrected images of the tomato fruit before lycopene extraction

Twenty tomatoes were processed, and lycopene was solvent extracted according to the protocol described in the methods section to generate their absorption spectra (Fig. 4A). The full spectrum was measured for tomatoes of different colours and at the wavelength of interest (503 nm) the absorbance peak increased as tomatoes ripened from green to red (Fig. 4B).

### Lycopene model generation and multi camera test

#### Model training

**Fig. 5:**
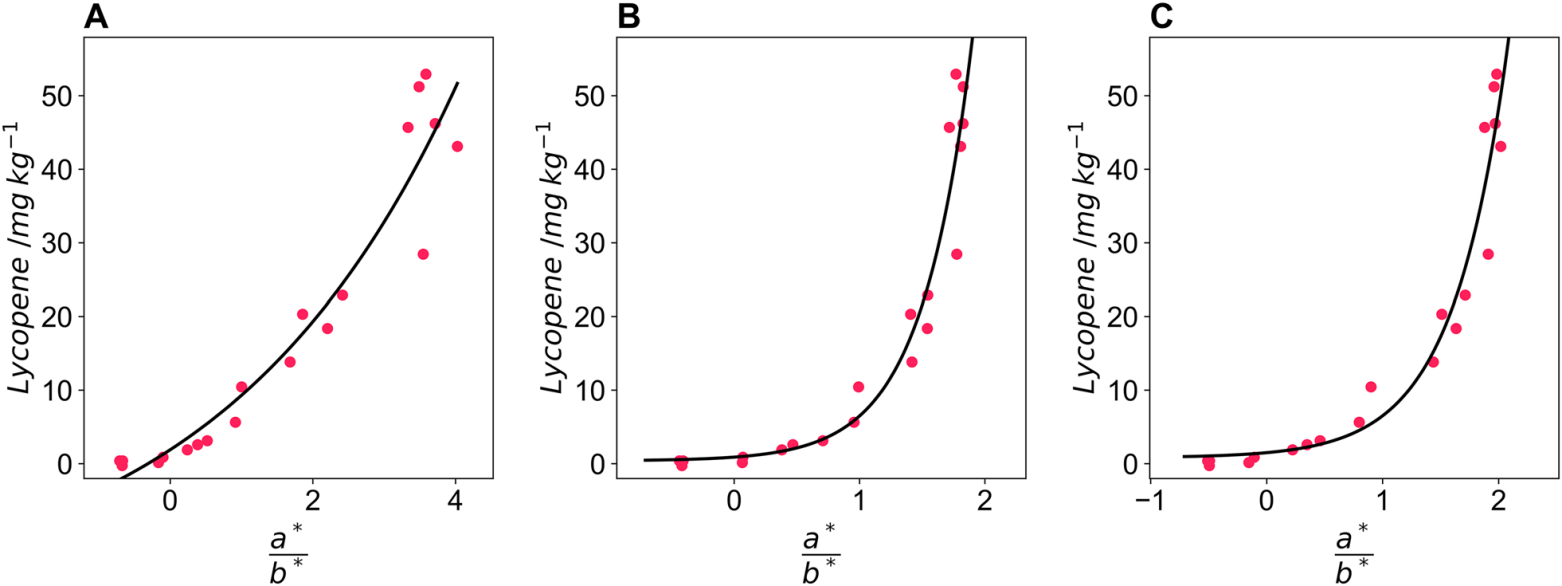
a*/b* values from Lab colour space of test tomatoes captured on different cameras; **A** Nikon D60, **B** Xioami Mi 9SE and **C** iPhone 7 against lycopene content determined via extraction and spectrophotometry. The black line indicates an exponential fit of the data used to predict lycopene content

**Fig. 6:**
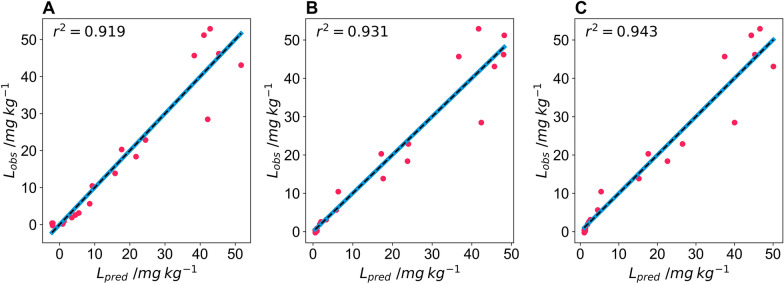
Predicted lycopene content determined from Lab colour space of test tomatoes captured on different cameras; **A** Nikon D60, **B** Xioami Mi 9SE and **C** iPhone 7 against lycopene content determined via extraction and spectrophotometry in mg lycopene per kg wet mass tomato. The black dashed line indicates the y = x line and the r^2^ of the fit is inset and the solid blue line shows the fit of the observed and predicted data

Twenty tomatoes were imaged using three different cameras: a DSLR camera (Nikon D60) and two mobile phone cameras (Xioami Mi 9SE and iPhone 7) to determine whether the pipeline is flexible in respect of the camera and camera settings used. The settings used for the DSLR camera were: aperture = f/4.2, shutter speed = 1/200 s, ISO = 100, file storage = RAW. For the two mobile phones, images were captured in automatic mode with no settings controlled. The images were processed using the colour and size pipeline, and Eq. (4) was used to model lycopene content using the CIELAB colour space. For all three cameras, the model proved successful, and suitable fitting parameters were determined for all three cameras (Fig. 5). The DSLR camera had the least amount of curvature of the three cameras, possibly due to proprietary image processing routines on the mobile phones increasing this curvature. The predicted lycopene values accurately predicted the observed lycopene values for the Nikon D60 (r^2^ = 0.919), Xioami Mi 9 SE (r^2^ = 0.931), and the iPhone 7 (r^2^ = 0.943) and the predicted against observed curve did not deviate from the y = x line for any of the cameras (Fig. 6).

The plant size and colour pipeline was able to successfully correct images from all three cameras and models with independent fitting parameters were developed for all three cameras. Although the DSLR camera that images in raw format has the model with the least curvature, all three models have high r^2^ indicating that the exponential model given in Eq. 4 is suitable for predicting lycopene content using the a*/b* ratio from the CIELAB colour space.

#### Model validation

**Fig. 7:**
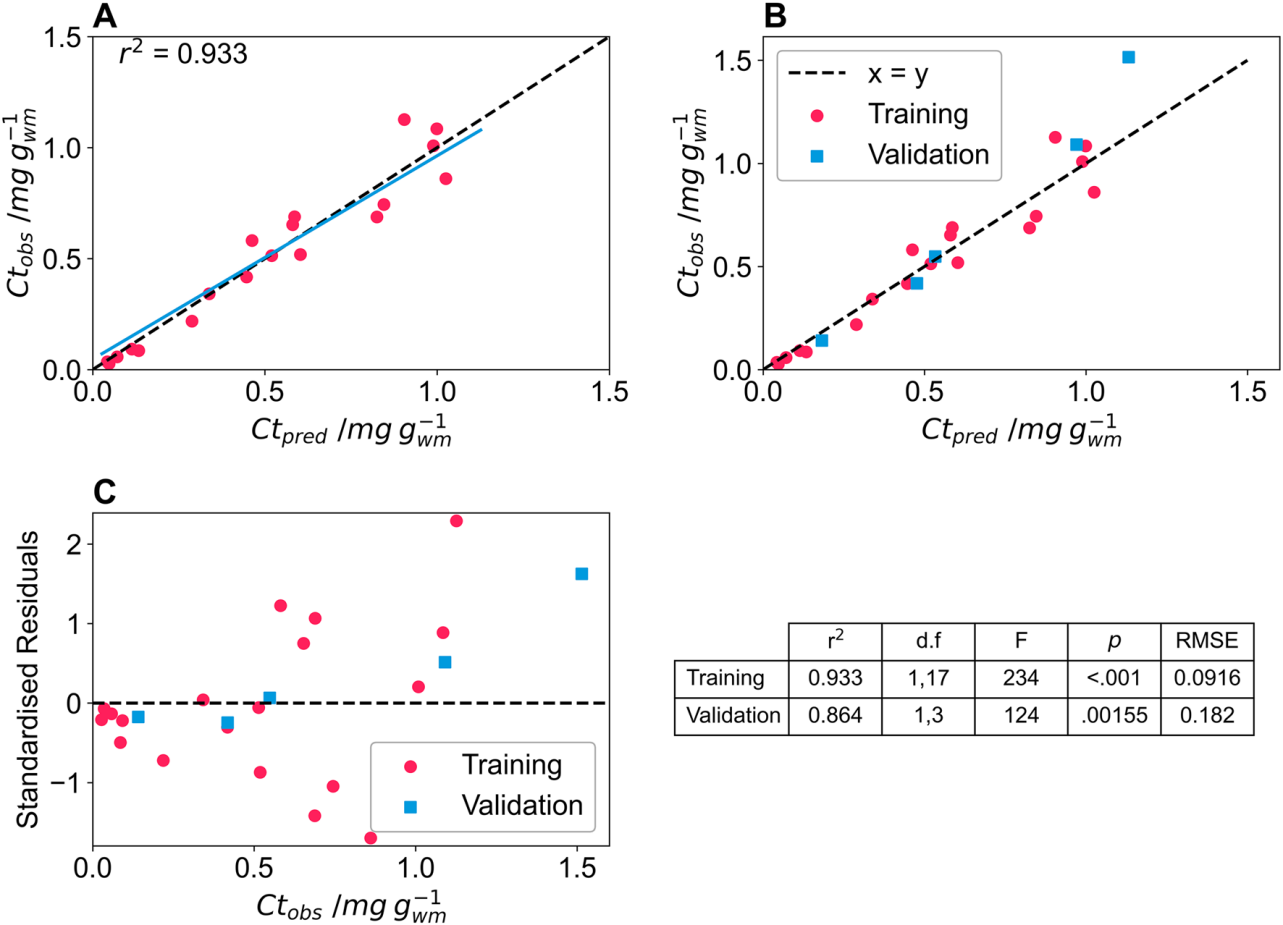
**A** a/b values from Lab colour space of test tomatoes captured on a Nikon D60 against lycopene content determined via extraction and spectrophotometry in mg lycopene per kg wet mass tomato. The solid line indicates an exponential fit of the data used to predict lycopene content. **B** Predicted lycopene content determined from Lab colour space of test tomatoes captured on Nikon D60 against lycopene content determined via extraction and spectrophotometry in mg lycopene per kg wet mass tomato. The dashed line indicates the y = x line. The pink circles show the data used to train the model and the blue squares indicate validation data points. **C** The standardised residuals of the training dataset, pink circles, and validation dataset, blue squares of the model fit and descriptive statistics of the model, with d.f; F and *p* indicating the degrees of freedom, F value and *p* value for an ANOVA of the predicted against observed values of the model for the training and validation datasets. RMSE is the root mean square error of the model

To validate the model, five more tomatoes were imaged and had their lycopene solvent extracted. This was done using only the Nikon D60 and images were taken inside the lightbox under the same lighting conditions as the training set. The lycopene content predicted from the a*/b* ratio was for all five tomatoes was within the expected range of the model (Fig. 7A). Furthermore, one of the five tomatoes in the validation set had a lycopene content much higher than any of the training tomatoes and the model accurately predicted this value (Fig. 7B). This was not the case for the models developed for the iPhone 7 (Additional file 8: S7). The standardised residuals for the model for training and validation show similar deviation for the two datasets (Fig. 7C) and an ANOVA of the predicted vs observed values for the training and validation set further highlight the precision of the model (Fig. 7C).

The validation of the a*/b* model shows that once these models are developed the pipeline, in conjunction with developed fitting parameters, can be used to predict the lycopene content of tomatoes. This validation set also indicates the importance of using a range of coloured tomatoes that cover the full range of expected colours to generate data over the entire range of lycopene content when training models, to avoid the need for extrapolating models outside of their training range. This is particularly important when using a mobile phone camera, where models have greater curvature.

### Chlorophyll extraction

**Fig. 8:**
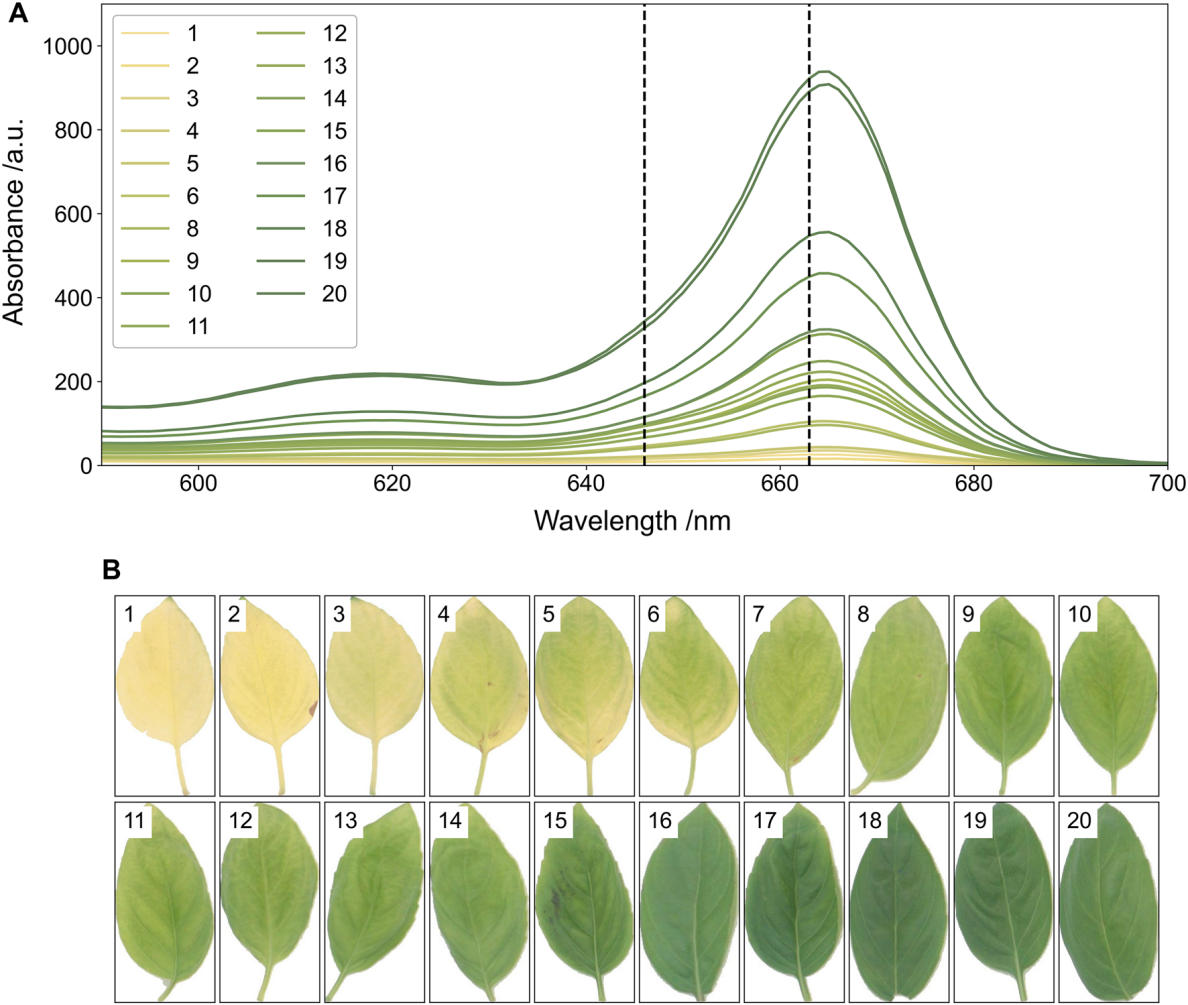
**A** UV spectrophotometry spectrum for basil leaves with the vertical dashed lines indicating the wavelengths used to determine total chlorophyll content (646 nm and 663 nm) and **B** background and colour corrected images of the basil leaves before chlorophyll extraction

Twenty basil leaves were processed, and chlorophyll was solvent extracted according to the protocol described in the methods section to generate their absorption spectra (Fig. 8A). The full spectrum was measured for leaves of different colours and at the wavelengths of interest (646 nm and 663 nm) the absorbance increased as the colour of the basil leaves changed from yellow to green (Fig. 8B).

### Chlorophyll model generation and validation

**Fig. 9:**
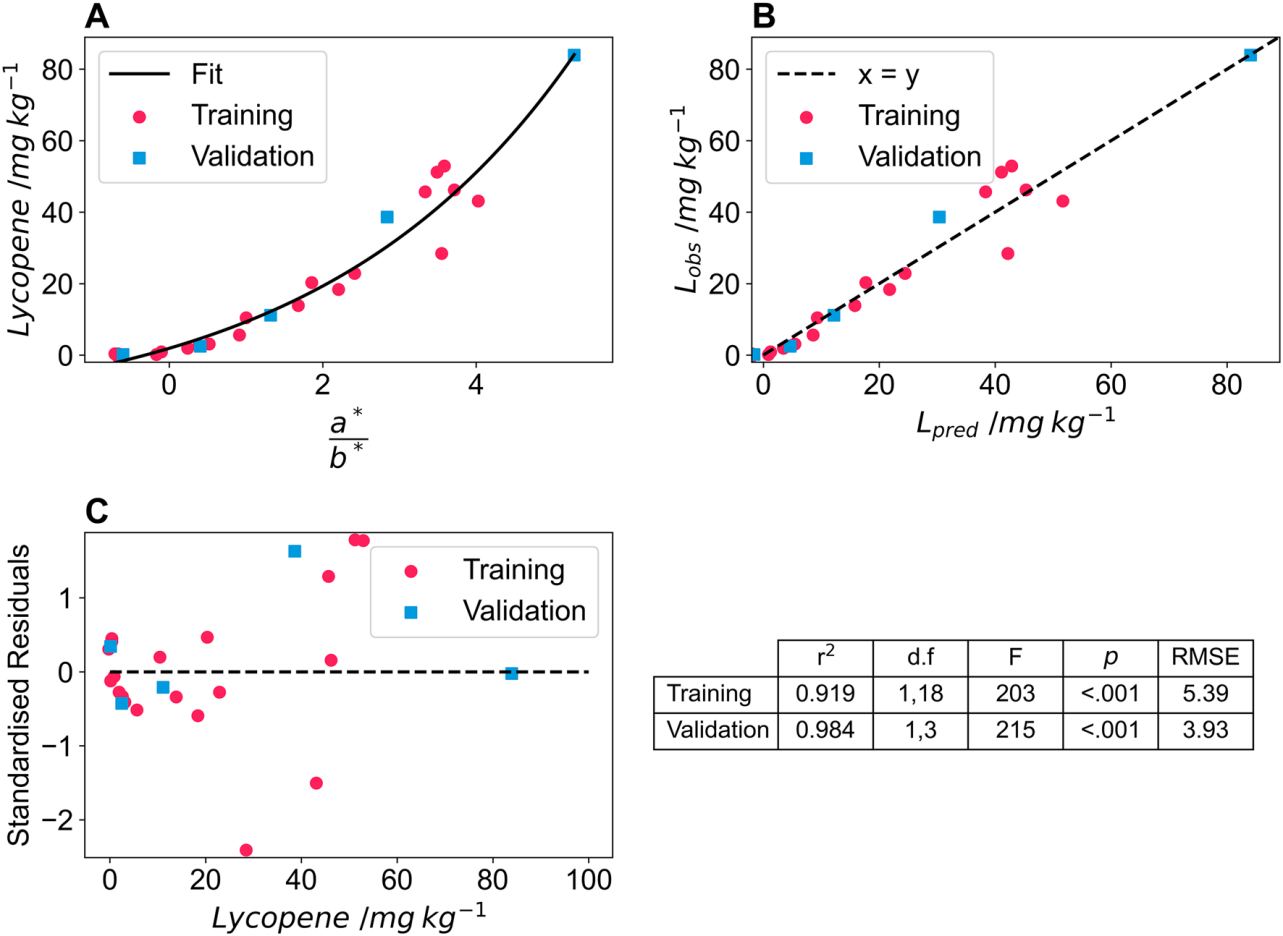
**A** Training data of predicted total chlorophyll content determined from RGB colour space of test basil leaves captured on a Nikon D60 against total chlorophyll content determined via extraction and spectrophotometry in µg chlorophyll per g wet mass basil. The r^2^ value is inset, the black dashed line indicates the y = x line and the blue line indicates the fit of the observed and actual data. **B** The blue squares show the validation data points predicted by the model and the dashed line indicates the y = x line. **C** The standardised residuals of the training dataset, pink circles, and validation dataset, blue squares of the model fit and descriptive statistics of the model, with d.f; F and *p* indicating the degrees of freedom, F value and *p* value for an ANOVA of the predicted against observed values of the model for the training and validation datasets. RMSE is the root mean square error of the model

Twenty basil leaves were imaged using a DSLR camera (Nikon D60). The settings used for the DSLR camera were: aperture = f/4.2; shutter speed = 1/200 s; ISO = 100; file storage = RAW. All images were taken inside a lightbox for the chlorophyll trials (both training and validation). The images were processed using the colour and size pipelines, and Eq. (8) was used to model chlorophyll content using the RGB colour space. Equation (8) was suitable for fitting colour data to the extracted chlorophyll content from basil leaves, as fitting parameters were found and the predicted values were similar to the observed values (r^2^ = 0.933). The predicted versus observed chlorophyll content curve deviated slightly from the y = x curve, indicating that the model slightly underpredicts chlorophyll content at low chlorophyll content and slightly overpredicts chlorophyll content at high chlorophyll content (Fig. 9A).

To validate the model, five more basil leaves were imaged and had chlorophyll extracted. Validation was done using only the Nikon D60, and images were taken inside the lightbox. The chlorophyll content was accurately predicted for four of the validation leaves; however, the fifth validation leaf had a chlorophyll content greater than any in the training set, and the chlorophyll content of this leaf was under predicted (Ct_pred_ = 1.13 µg g^−1^; Ct_obs_ = 1.51 µg g^−1^) (Fig. 9B). The standardised residuals for the model for training and validation show similar deviation for the two datasets (Fig. 9C). An ANOVA of the predicted vs observed values for the training and validation dataset showed a lower F and *p* value for the validation dataset likely due to the single data point from outside of the training range skewing the result (Fig. 9C). This point is left in the analysis to highlight the importance of training models using a subset of leaves that cover the full range of expected chlorophyll content.

This underprediction in the validation data set reinforces what we learned from the lycopene validation data. In order to use colour data to accurately predict coloured compounds in plant materials, the training set needs to incorporate samples from the entire expected colour range, as model prediction is only accurate within the range of the training data (it does not always extrapolate accurately). This is particularly important when models have large curvature, such as the two models generated using the mobile phone cameras for lycopene content.

**Fig. 10:**
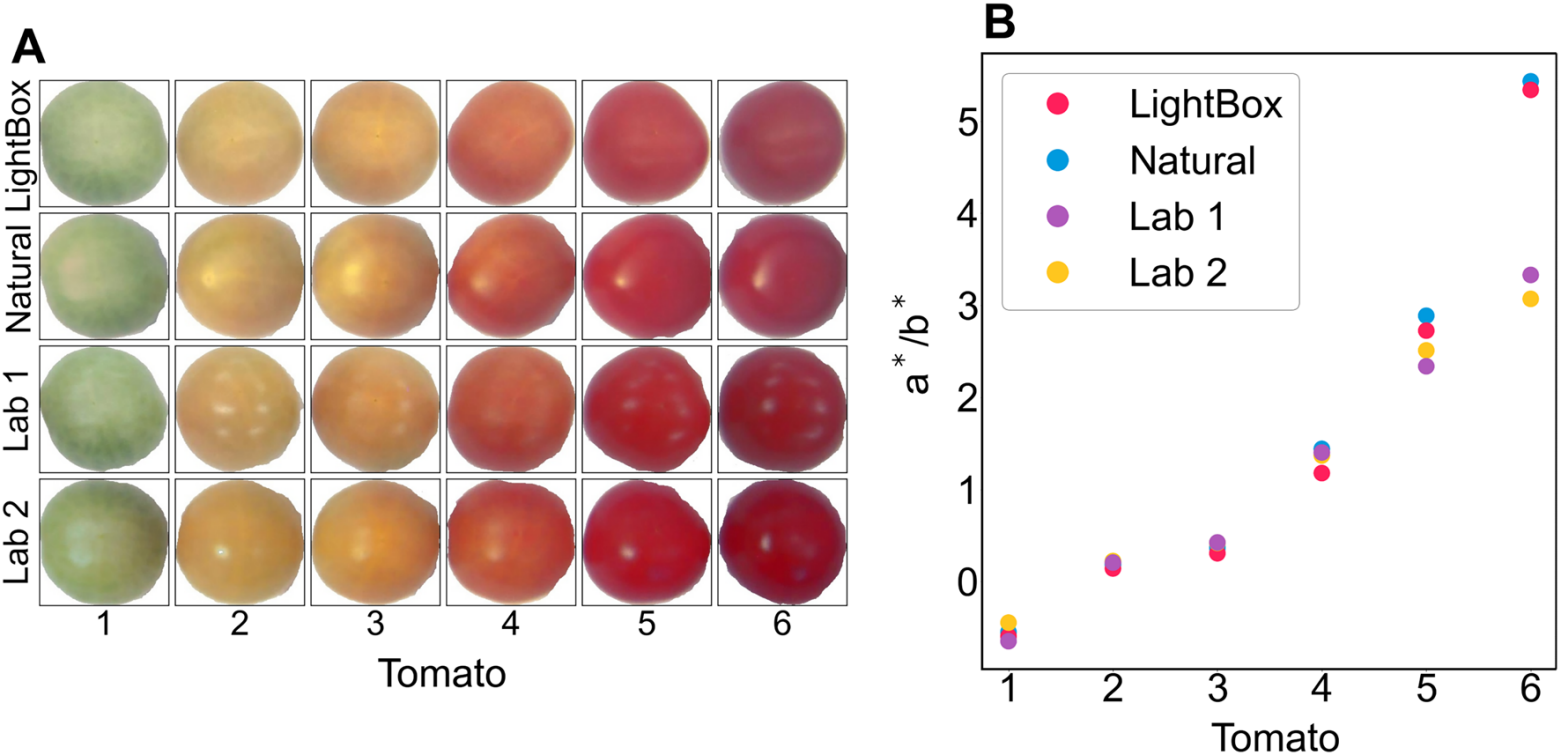
**A** The six validation tomatoes captured under four different lighting conditions after colour correction and **B** the calculated mean a*/b* for the six tomatoes under the four lighting conditions

Validation tomatoes were also captured under the four different lighting conditions (Fig. 10A) used in the lighting test to determine importance of consistent lighting when capturing images (using the Nikon with the same capture settings as all previous captures). The a*/b* value is consistent for the four lighting conditions for low a*/b* (and lycopene) values, however for tomato 5 and tomato 6 the a*/b* value is significantly different between the lightbox/natural lit images and the two fluorescently lit images, lab 1 and lab 2 (Fig. 10B). This is likely due to the substantial amount of light reflected in lab 1 and lab 2. This result suggests that lighting should be kept as consistent as possible when capturing images for quantification of chemical compounds by colour. Furthermore models developed under one set of lighting are unlikely to be transferrable if lighting is drastically changed.

## Conclusions

A set of open source Python scripts is presented with a pipeline for imaging, background correction, colour correction, cropping, separating objects, and determining the size and colour of objects. The pipeline is validated using lycopene extraction from tomatoes and chlorophyll extraction from basil leaves. Tomatoes were imaged in four different locations under four different lighting conditions (lightbox, natural light, and two different fluorescent lights) using three different cameras (a DSLR and two mobile phones). In each of these cases, a colour corrector was imaged, and the difference between the actual colour of the swatches and the captured colour was compared. The error was highest before colour correction in the images captured under fluorescent lighting conditions and was lowest in natural light and in the lightbox. Colour correction reduced this error in all locations, and there was no difference in the error of the swatches between the four locations after colour correction. All three cameras were able to predict lycopene content accurately; however, validation indicated that the DSLR camera was the only one that accurately predicted lycopene content outside of the training set. Furthermore, tomatoes captured under different lighting conditions, even after colour correction, had different mean a*/b* values at high lycopene content. The chlorophyll content of basil leaves was accurately predicted using the DSLR camera in the lightbox; however, validation showed that the model underpredicted when a leaf was imaged from outside the training set. This indicates that model training when imaging plant materials should include the entire range of expected plant material colours to ensure accurate predictions.

### Supplementary Information

**Additional file 1: **List of python packages required for pipeline.

**Additional file 2: **Example background image.

**Additional file 3: **Example of capturing image.

**Additional file 4: **RGB spyderCheckr24 data.

**Additional file 5: **Example for generating swatch file.

**Additional file 6: **Light spectrums for the four locations.

**Additional file 7: **Untransformed colour correction data.

**Additional file 8: **Model validation for tomatoes captured on iPhone.

## Data Availability

All data generated for this manuscript is available at: http://www.doi.org/10.15131/shef.data.21989561. The project snapshot is hosted at http://www.doi.org/10.17605/OSF.IO/QAYMU and the up to date script files are available at: https://github.com/HarryCWright/PlantSizeClr.
