# Supplementary figures and images for: Free and open-source software for object detection, size, and colour determination for use in plant phenotyping
Source: Plant Methods. 2023 Nov 15;19:126. doi: 10.1186/s13007-023-01103-0 (PMC10647133; doi:10.1186/s13007-023-01103-0)

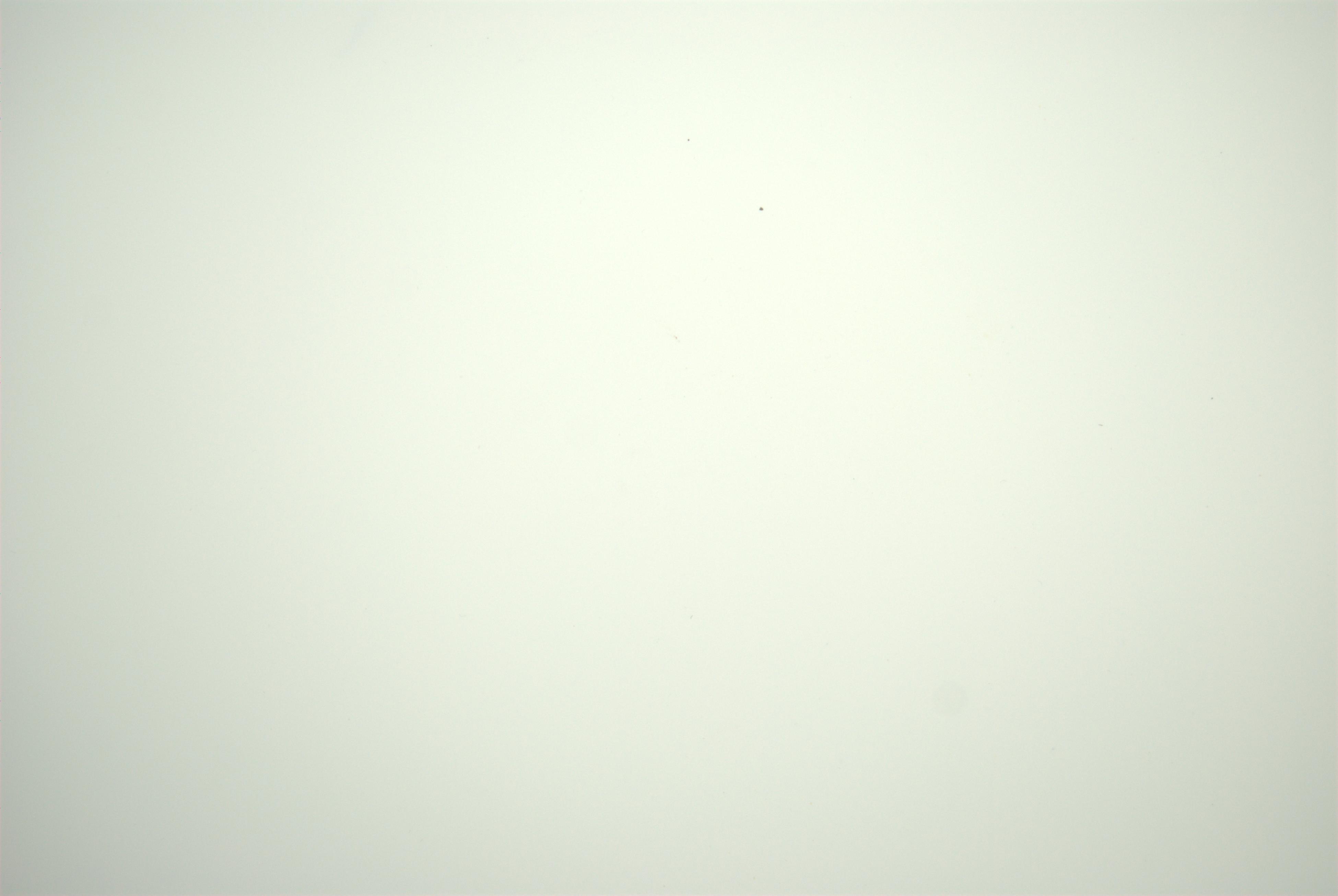

Supplement: Supplementary file 2 — Additional file 2: Example background image. [file 13007_2023_1103_MOESM2_ESM.jpg]

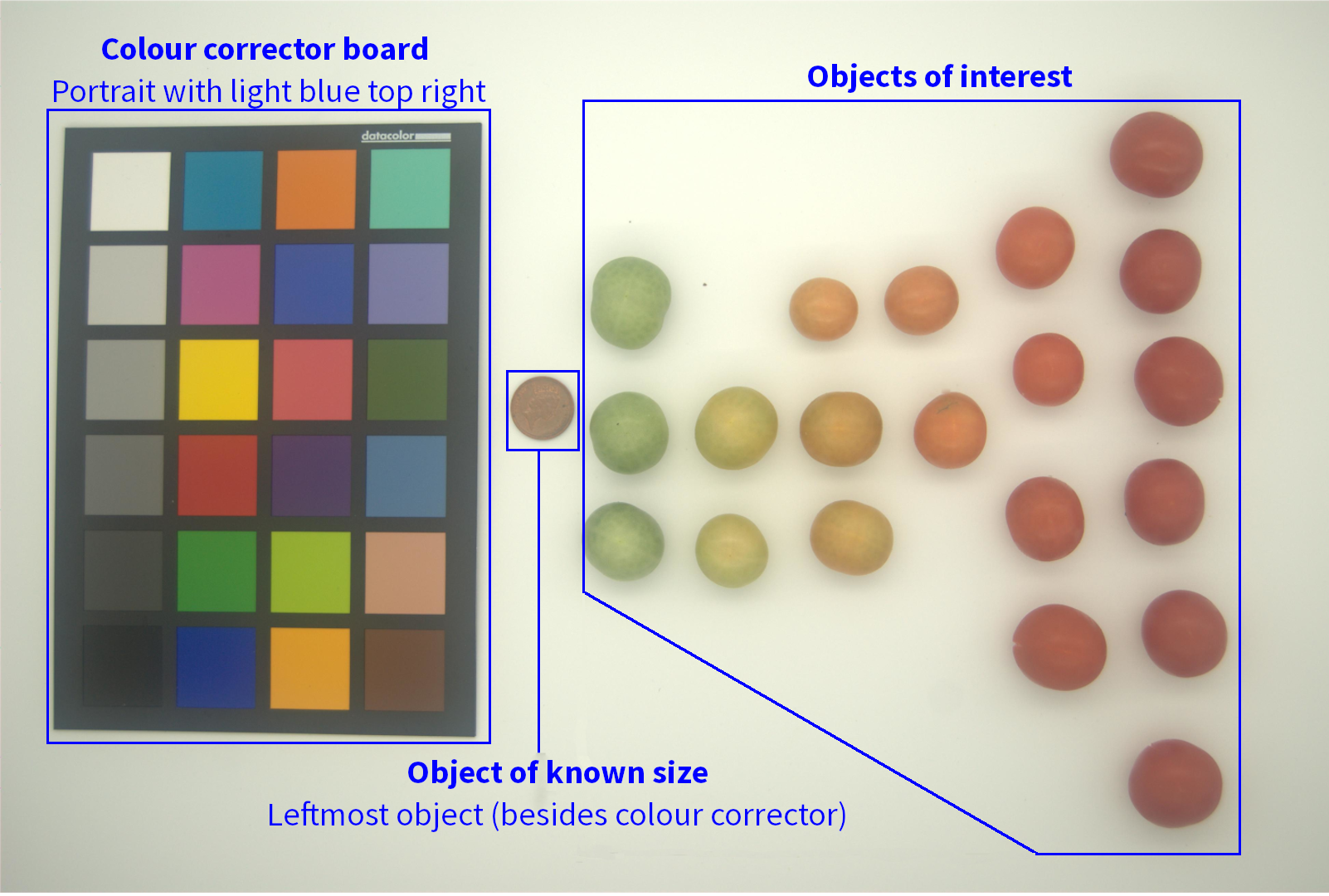

Supplement: Supplementary file 3 — Additional file 3: Example of capturing image. [file 13007_2023_1103_MOESM3_ESM.png]

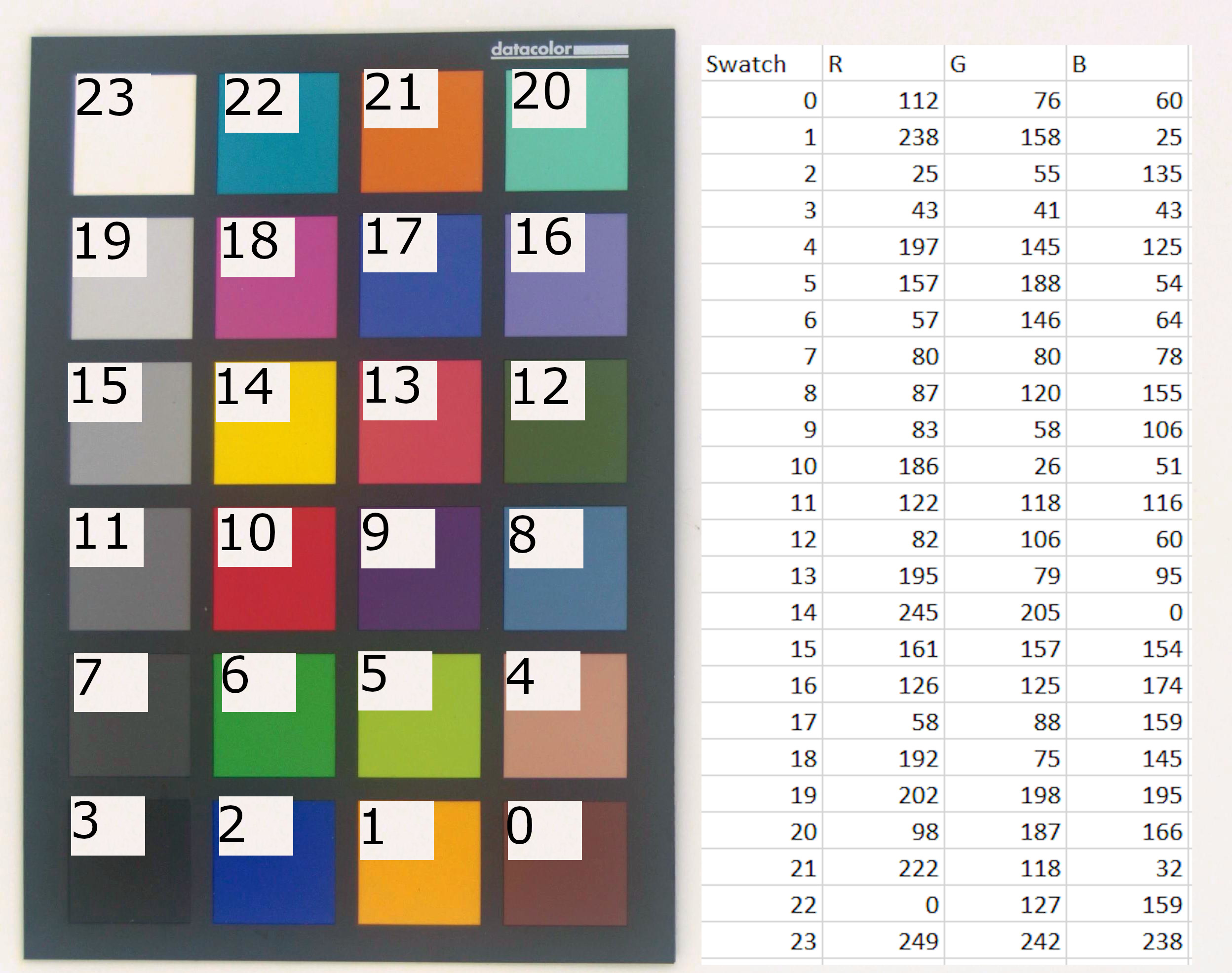

Supplement: Supplementary file 5 — Additional file 5: Example for generating swatch file. [file 13007_2023_1103_MOESM5_ESM.jpg]

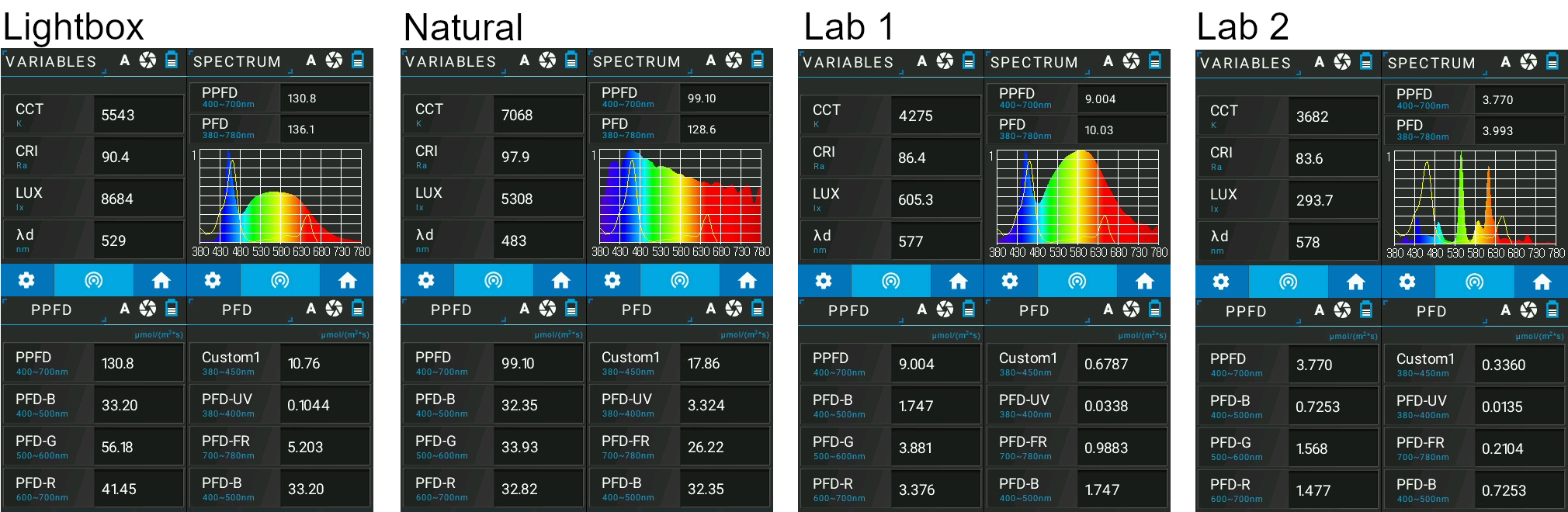

Supplement: Supplementary file 6 — Additional file 6: Light spectrums for the four locations. [file 13007_2023_1103_MOESM6_ESM.png]

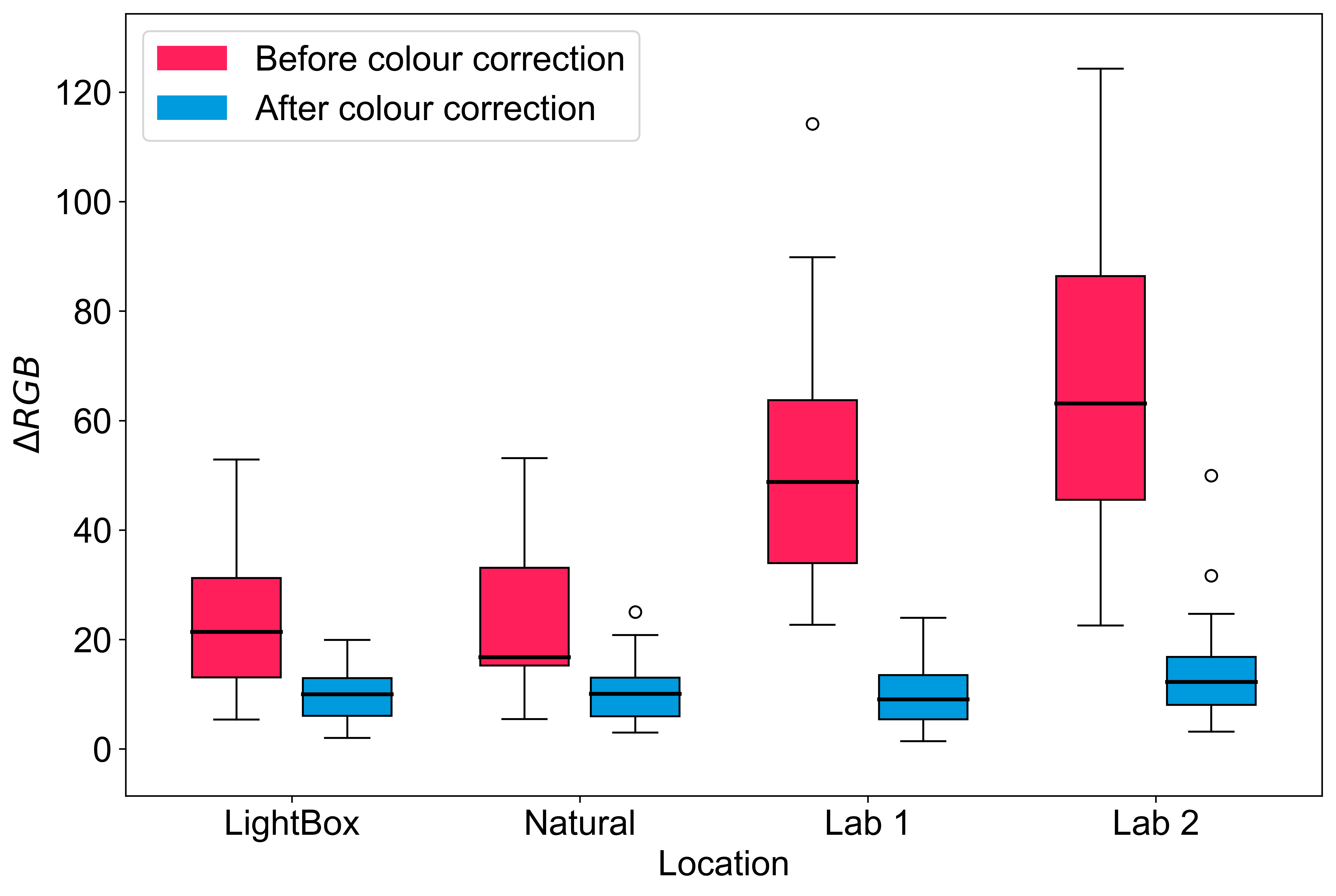

Supplement: Supplementary file 7 — Additional file 7: Untransformed colour correction data. [file 13007_2023_1103_MOESM7_ESM.png]

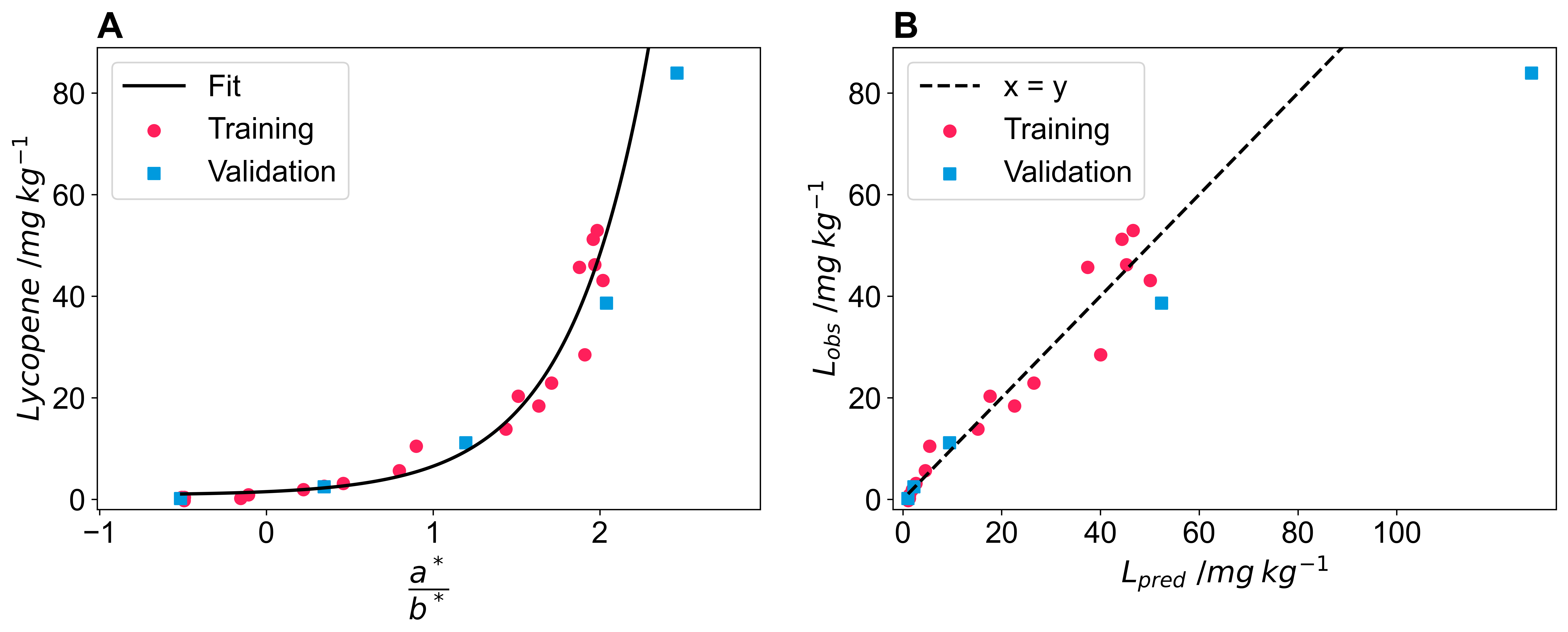

Supplement: Supplementary file 8 — Additional file 8: Model validation for tomatoes captured on iPhone. [file 13007_2023_1103_MOESM8_ESM.png]
